# The mediating role of psychological resilience between social participation and life satisfaction among older adults in China

**DOI:** 10.1186/s12877-022-03635-x

**Published:** 2022-12-08

**Authors:** Zhiliu Liao, Hanmeng Zhou, Zhifei He

**Affiliations:** 1 School of Public Affairs, Fujian Province, Xiamen University, Xiamen City, People’s Republic of China; 2 School of Politics and Public Administration, Southwest University of Political Science and Law, Chongqing, People’s Republic of China

**Keywords:** Older adults, Social participation, Psychological resilience, Life satisfaction, Mediating effect

## Abstract

**Background:**

A significant correlation has been discovered between social participation and older adults’ life satisfaction, but the relationships among social participation, psychological resilience, and life satisfaction remain to be confirmed. Therefore, this study aims to identify the relationship between social participation and life satisfaction for Chinese older adults and to analyse the possible mediating role of psychological resilience between these two aspects.

**Methods:**

Data on 15,779 people aged 65 years and above were extracted from the 2018 wave of the Chinese Longitudinal Healthy Longevity Survey (CLHLS). Social participation was classified into two levels: low-level involvement activities and high-level involvement activities. Hierarchical regression analysis was applied to analyse the correlations between the two levels of social participation and older adults’ life satisfaction as well as the mediating effects of psychological resilience on this association.

**Results:**

The results indicate that two levels of social participation were each positively correlated with life satisfaction. Specifically, high-level involvement activities (*β* = 0.070, *P* < 0.001) were more strongly associated with life satisfaction than low-level involvement activities (*β* = 0.051, *P* < 0.001). Moreover, psychological resilience was found to partially mediate the association between low-level involvement activities and high-level involvement activities and life satisfaction.

**Conclusion:**

A higher level of life satisfaction for older adults is related to participation in high-level involvement activities. Psychological resilience has a mediating effect on the association between two levels of older adults’ social participation and life satisfaction. These findings suggest that the government and society should establish a more concrete understanding of the psychological resilience of older adults.

## Background

Globally, the population of older persons aged 60 years and above will be 1.4 billion in 2030 and 2.1 billion in 2050 [1]. China faces challenges from an ageing society, and the latest population distribution in China shows that 14.2% of the total population was aged 65 years and above in 2021 [2]. Older adulthood is a life stage associated with many uncertain psychosocial and biological changes, which may lead to a decline in subjective well-being. One of the most important indicators of subjective well-being is life satisfaction [3]. Older adults’ life satisfaction is frequently thought to decrease gradually with age due to increasing dependency, health problems, loss of close relationships [4], and individuals approaching death [5]. Therefore, it is critical to explore the aspects that may contribute to life satisfaction.

### Social participation and life satisfaction

One of the factors that influences life satisfaction is social participation, which is generally viewed as individuals’ participation in activities that involve interactions with others [6] as well as fulfilling daily activities and more valuable social roles for individuals and society [7]. Aspects of social participation include activities that involve active personal engagement (e.g., travelling), certain economic value, such as employment and paid work, leisure activities, social activities, and housework [8, 9] as well as virtual activities that connect individuals to certain social groups (e.g., watching TV, participating in internet forums) [10]. Improving social participation is the essential proposal of the World Health Organization’s policy on the ageing population [11]. To have an active and successful ageing population, it is essential to encourage older persons to participate in social activities. Some previous studies have indicated a positive correlation between social participation and the improvement of life satisfaction among older adults [12, 13]. For instance, attendance in any form of social activities was positively connected to life satisfaction among Chilean older persons [14]. Another study revealed that older adults are less active in social engagements because of their deteriorating physical condition, which results in worse quality of life [15]. However, older adults place a high value on social participation, which brings them fulfilment. Social participation promotes a nonsedentary lifestyle that includes exercise and physical activity, which may assist older adults in keeping their minds sharp [16]. Older people consciously and actively engage in outdoor social activities to interact and share resources with others and achieve a satisfactory life as a result [17]. Previous research has shown that older adults’ engagement in leisure activities is positively correlated with happiness and that older adults experience social satisfaction by taking part in various group activities [18]. A survey of Croatian older people indicated that participating in leisure activities may enhance subjective well-being [19]. By participating in leisure activities, older adults can build social relationships, fulfill life values, establish positive emotions and improve subjective well-being [20]. Another study showed that membership in multiple social organizations is related to high life satisfaction, possibly because older adults can participate in social activities and maintain certain social roles and status, which increases their happiness [21]. Social participation differs between older and younger groups because the former group shifts their focus away from work after retirement [6]. Due to cultural differences, common types of social activities might differ in China compared to other areas [22]. Social activities such as playing cards or mahjong are the most popular activities among older adults in China [23], while opportunities for formal events hosted by social organizations, such as charity work or voluntary services, are rather limited [24]. However, playing cards or mahjong or attending organized social events incorporates social engagement and helps Chinese older persons accommodate their life changes [25]. Additionally, accumulating evidence has indicated that participating in activities such as watching TV, reading, surfing the internet and listening to the radio represent less interactive forms of social participation in which older adults are passively exposed to social information and news and can contribute to life satisfaction [26–28]. Social participation differs in various cultural and social contexts. Housework activities, such as cooking and taking care of children, are common phenomena in many Asian countries. It is also essential to encourage older adults to expand their contacts with their children, other generations of relatives and other older persons of different ages. The level of happiness and life satisfaction is higher among older adults who engage in housekeeping chores [29]. Furthermore, older adults participate in different levels of social activities, and the individuals’ interactions with others may produce different levels of life satisfaction. A previous study found that older people with lower engagement in enjoyable activities had more negative moods and a more negative affect than those with higher engagement [30]. Additional research has shown that social activities outside the home have a greater impact on life satisfaction among older adults [31]. Although the direct impact of participation in social activities on older people’s life satisfaction has been widely explored, previous studies have used aggregated indicators of social participation [32] or focused on one of its particular forms. However, the underlying mechanisms by which different levels of social participation impact life satisfaction among Chinese older adults are not well examined. As previous research suggests, it is critical for people of all ages to develop and maintain social participation, including older adults [33]. The rate of social participation of older adults in China is relatively lower than that of their peers from Western nations [34]. Therefore, our study explores the effect of different levels of social participation on the life satisfaction of Chinese older adults to promote active and successful ageing in China.

### Psychological resilience and life satisfaction

Another factor closely related to older adults’ life satisfaction is psychological resilience. Psychological resilience is a positive mental asset that is considered to be a critical protective mechanism for individuals by making them capable of recovering from stress or adversity [35]. The presence of psychological resilience is significantly correlated with a higher level of life satisfaction throughout the human lifespan [36]. Previous literature on psychological resilience has primarily paid attention to the early period of life, specifically childhood and adolescence [37]. However, there is evidence that psychological resilience may occur at any stage in the human lifespan [38]. Therefore, psychological resilience is important in the later life stages. As a psychological asset, resilience contributes to older adults’ quality of life, mental health, ageing success and reduction in mortality risk [39, 40]. The empirical literature shows that resilience is strongly associated with life outcomes and improves life satisfaction [41]. However, few studies have explored the correlations between psychological resilience and older people’s life satisfaction [42]. A nomadic study showed that life satisfaction and resilience were positively associated among older adults [43]. Older adults who are more psychologically resilient may be more capable of overcoming mental health illnesses and physical health challenges.

### Social participation and psychological resilience

People’s psychological resilience is also associated with social participation. Social participation within the community has frequently been found to be related to psychological resilience among older people [44]. Psychological resilience refers to the ability to adapt to life and environmental changes; when older adults are more psychologically resilient, they also show strong enthusiasm for participating in activities [45]. According to Baltes et al. [46], when older people experience a loss of psychological, physical and social resources, they can mitigate the effects of these factors by utilizing their own, family and community resources as well as other protective factors in one of three ways: selection, optimization, or compensation (SOC) [47]. Consequently, they are better able to adapt to their new situation and lead more rewarding lives. Older adults with high psychological resilience may rely on their own psychological strength to overcome adversity and achieve happiness, whereas older adults with low psychological resilience may require assistance from other resources to resist the negative impact of adverse factors [48]. Social participation, as an external resource, can compensate for a lack of psychological resilience, strengthen older adults’ perception of psychological resilience, and ultimately improve their life satisfaction [49]. Simultaneously, psychological resilience can be promoted and strengthened through social participation. A previous study showed that older adults with a high level of social participation often have a higher level of psychological resilience [50]. A high degree of social participation decreases the likelihood of mental illness risks because social participation may enhance the physiological health of older adults, such as boosting host resistance, buffering stress, and lowering the biomarkers of disease risks [51]. Social and spiritual support leads to a reduction in mental health illness risks and an increase in psychological resilience in this population. Activities such as sports and travel can increase the intrinsic motivation of older adults and thus improve their mental health [52]. Participating in social events, such as voluntary events, can improve individuals’ mental health through the establishment of social connections and self-motivations [53]. The majority of older people in China babysit their grandchildren because of the traditional concept of family. Previous research shows that raising grandchildren significantly improves the mental health of older persons in China [54]. Although some previous studies have explored the relationship between social participation and psychological resilience and between psychological resilience and life satisfaction, there is still a lack of research on the impact of psychological resilience on social participation and life satisfaction among older adults. Therefore, this paper also aims to explore the associations among social participation, psychological resilience and the life satisfaction of older adults in China.

**Fig. 1:**
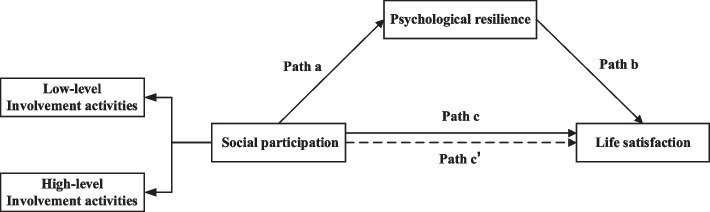
Hypothetical model.Notes:path a, effect of social participation on psychological resilience; path b, effect of psychological resilience on life satisfaction; path c, total effect of social participation on life satisfaction; path c’, direct effect of social participation on life satisfaction when psychological resilience as a mediator

Activity theory is a psychosocial theory of ageing that aims to define individuals and social life conditions that maximize happiness and satisfaction [55]. Activity theory focuses on the relationships among activity participation, quality of life and life satisfaction [55, 56]. Based on activity theory, staying active in old age can offset the role loss faced by older adults after retirement, maintain the balance formed in middle age, improve quality of life and postpone the ageing process [57]. In lifespan developmental research, especially among older adults, the SOC model is frequently used within an action-theoretical framework. The SOC model encourages adaptive functioning and development throughout the lifespan, and resource-maintaining and loss-minimizing compensatory and loss-based selection actions may become more significant in older adulthood [58]. Thus, the associations between social participation, psychological resilience, and life satisfaction for older adults can be theoretically supported by activity theory and the SOC model. This study developed a conceptual framework based on the theoretical and empirical basis provided above (Fig. 1) with two hypotheses: H1) Social participation, including low-level involvement activities and high-level involvement activities, is positively related to the life satisfaction of older adults, and high-level involvement activities are more strongly associated with life satisfaction than low-level involvement activities. H2) Psychological resilience mediates the association between social participation and life satisfaction.

## Methods

### Data source

The data of the population aged 65 years and above were extracted from the 2018 wave of the Chinese Longitudinal Healthy Longevity Survey (CLHLS). With a stratified multistage cluster sampling methodology, the CLHLS randomly chooses a sample of rural and urban older adults throughout 22 provinces, representing 85% of China’s total population. The ongoing national representative longitudinal survey began in 1998, with the survey population initially consisting of older adults aged 80 and over. It began interviewing younger-old respondents (65 years old and over) in 2002. In 2002, 2005, 2008, 2011, 2014 and 2018, the CLHLS conducted follow-up surveys and recruited new respondents, and the response rate of each survey was approximately 90% [59]. There were approximately 16,064 participants who were 65 or older in the 2002 baseline follow-up surveys of the CLHLS. Among them, 2,015 (12.54%), 1,464 (9.11%), 494 (3.08%), 116 (0.72%), and 408 (2.54%) were lost in the follow-up waves of 2005, 2008, 2011, 2014 and 2018, respectively, while 5,874 (36.56%), 2,520 (15.69%), 1,184 (7.37%), 717 (4.46%), and 484 (3.01%) respondents died before being interviewed in the follow-up waves. Our study used the 2018 wave of CLHLS data, which interviewed 15,874 older Chinese citizens, and 12,411 (78.18%) respondents were interviewed for the first time. The dataset includes sociodemographic characteristics, physical and mental health status, social activities, cognitive ability and so on. CLHLS data are credible nationally representative longitudinal data and comprise numerous older adults in China [60]. CLHLS has conducted overproportional sampling on older adults and male and urban older adults in the sample design. Since age, gender and urban and rural variables were controlled in the model, weighting methodology is not necessary to apply here [61]. The 2018 wave survey contains some data incompleteness because older adults frequently respond to questions about feelings, attitudes and expectations with “don’t know” and “missing” responses [62]. To analyse the data effectively, when the incomplete rate exceeded 0.50%, we employed multiple imputation to obtain estimates for missing values [63]. There were no missing values for gender or current residence, while the proportions of missing values were approximately 10% or higher: chronic disease (21.57%), psychological resilience (16.17%), education (14.94%) and life satisfaction (9.06%). The remaining missing values were all less than 3%. Ninety-five persons under the age of 65 were eliminated, and we paid particular attention to the subsample of Chinese older adults aged 65 and over. The final sample contained 15,779 Chinese older adults.

### Measures

#### Variables

##### Life satisfaction

Life satisfaction is generally defined as an individual’s emotional and cognitive assessment of life and reflects standards to measure quality of life [64]. This study used the following question in the CLHLS to measure the life satisfaction of older adults: “How do you rate your life at present?” We reverted the original five-point Likert scale to 1 (very bad), 2 (bad), 3 (‘so so’), 4 (good), and 5 (very good). A higher score indicates greater holistic life satisfaction.

##### Social participation

The key explanatory variable was social participation. A total of 10 social participation activities were measured in this study, including housework, outdoor activities, raising domestic animals, gardening, watching TV and/or listening to the radio, organizing social activities, reading newspapers or books, playing cards/mahjong, doing exercises regularly and travelling. Because 1–8 items of the social participation activities were continuous variables, five choices for each activity were given to the participants: 1 (almost every day), 2 (not every day, but at least once a week), and 3 (not every week, but at least once a month). 4 (not every month, but sometimes), and 5 (never). Dichotomous variables were used for 9–10 items of the social participation activities. Consistent with previous studies [53], all variables were dichotomized into “yes” (= 1) or “no” (= 0). Furthermore, 1–8 items of activities were recoded to a dichotomous variable that represented whether older adults participated in the activity. Exercising regularly was a measure of a dichotomous variable reflecting older adults’ involvement in physical exercise (coded as 1) or not (coded as 0). According to previous studies [65], travel time was measured by a dichotomous variable (coded 0 for those who had no tourism experience within the past two years and coded 1 for those who had at least one tourism experience).

There is no standard and unified classification system for social participation, and there are numerous ways to measure it [66]. Levasseur et al. [6] classified social participation into six levels ranging from low to high based on the individual’s level of involvement with others (level 1: alone, level 2: parallel, levels 3–6: interaction). All daily activities that a person often performs by themselves are at level 1 in preparation for activities that will interact with other people. These are fundamental and survival activities, such as preparing meals or housework. Level 1 also includes solitary activities such as listening to the radio and watching television; these activities keep people informed about what is going on in society, which is a common way to initiate conversation with others. The first-level activities are typically performed by people alone and at home. Activities at level 2 include those in which the individual is not directly in contact with others but is surrounded by others. Therefore, people who participate in social activities at levels 1–2 are alone and have little or no interaction with others. Their daily activities primarily take place at home or in the community. Significant interpersonal engagement occurs at levels 3–6 of social participation. Given the CLHLS database limitation and the social context and cultural preferences for activities among older persons in China, our study sorted the items based on Levasseur et al.’s levels of involvement with others: activities with little or no interaction with others were considered a “low level” (level 1–2), and activities with higher interaction were considered a “high level” (level 3–6). In this study, social participation was classified into low-level involvement activities and high-level involvement activities. Low-level involvement activities include watching TV and/or listening to the radio, reading newspapers/books, raising domestic animals, gardening and housework, with a continuous variable ranging from 0 to 5. High-level involvement activities include outdoor activities, travelling, exercising, playing cards and/or mahjong and organized social activities, with a continuous variable ranging from 0 to 5. A higher score represents better engagement in social participation. Details concerning the classification of social participation are presented in Table 1.

**Table 1:** Classification of social participation

Level	Items
Low-level involvement activities	Watch TV and/or listen to radio
	Read newspapers/books
	Garden work
	Housework
	Raise domestic animals
High-level involvement activities	Outdoor activities
	Travel in the past two years
	Do exercises
	Play cards and/or mahjong
	Organized social activities

##### Psychological resilience

The key mediation variable is psychological resilience. Consistent with measures used in previous research [40, 63], psychological resilience was examined by asking older adults five questions in the CLHLS: 1) Do you always look on the bright side of things? 2) Do you feel the older you get, the more useless you are and do you have trouble doing anything? 3) Do you often feel lonely and isolated? 4) Do you often feel fearful or anxious? 5) Can you make your own decisions concerning your personal affairs?The values ranged from 1 to 5 (1 = always; 2 = often; 3 = sometimes; 4 = seldom; 5 = never). Items 1 and 5 were reverse recoded. Five questions were summed to obtain a total psychological resilience score for each older person; scores ranged from 6 to 25, with higher scores representing better psychological resilience. The CLHLS did not mainly collect data to investigate the psychological resilience of older adults, and the above measures may not be perfect indicators of older adults’ psychological resilience. However, Yang et al. [63] indicated that these measures represent important dimensions of psychological resilience. Cronbach’s alpha value for psychological resilience in this study was 0.89.

##### Covariates

Based on previous studies [25, 67], the control variables were divided into the following three categories: sociodemographic characteristics, socioeconomic status (SES) and health status. The first group of control variables consisted of the respondents’ sociodemographic characteristics, including gender (female = 1), age, and current marital status (married = 1). Current residence was classified as rural or urban (urban coded as 1). Age was divided into three life-stage subgroups (ages 65–74, ages 75–84 and ages 85 and above). The second group of covariates consisted of socioeconomic status with two variables. Education level was classified as literate (coded as 1) or illiterate (coded as 0). Financial resources were measured by respondents’ answers of whether their financial support was sufficient for daily costs (sufficient coded as 1). Chronic disease depended on whether respondents had a chronic disease or not (yes coded as 1). The analysis also controlled for older adults’ living arrangements. These factors have been found to influence life satisfaction. Table 2 provides a description of the study variables.

**Table 2:** Descriptive statistics of the sample’s characteristics (*N* = 15,779)

**Variables**	**Frequency**	**%**
**Age**		
65–74	3282	20.80
75–84	4277	27.11
≥ 85	8220	52.09
**Sex**		
Male	6873	43.56
Female	8906	56.44
**Marital status**		
Married	6450	40.88
Widowed, never married and divorced	9329	59.12
**Education**		
Illiterate	7961	50.45
Literate	7818	49.55
**Current residence**		
Urban	8738	55.38
Rural	7041	44.62
**Living status**		
With family	12,684	80.39
Alone	2514	15.93
Institution	581	3.68
**Financial support**		
Yes (enough)	13,507	85.60
No (not enough)	2272	14.40
**Chronic disease**		
Yes	11,016	69.81
No	4763	30.19
**Variables**	**Mean**	**SD**
Low-level involvement activities [0–5]	1.796	1.324
High-level involvement activities [0–5]	1.329	1.219
Psychological resilience[6–25]	19.160	3.100
Life satisfaction[1–5]	3.883	0.797

Note. The minimum score and maximum score of every continuous are present in brackets

##### Statistical analysis

Descriptive analysis was conducted to identify the participants’ characteristic distribution. Frequencies and percentages described categorical variables, while statistical means and standard deviations measured continuous variables. The association between correlation analyses was calculated for low-level involvement activities and high-level involvement activities, psychological resilience and life satisfaction. The Pearson *χ*^*2*^ test was applied to test the significance of differences among psychological resilience. We followed Baron et al.’s [68] method to identify relationships among social participation, psychological resilience and life satisfaction based on hierarchical regression analysis. First, linear regression was applied to predict the correlation between independent variables (low-level involvement activities and high-level involvement activities) and the dependent variable (life satisfaction) (test c). Second, regression analysis was performed by independent variables (low-level involvement activities and high-level involvement activities) to predict the mediating variable (psychological resilience) (test a). Third, multiple regression analysis was examined with the mediating variable (psychological resilience) and independent variable to predict the dependent variable (life satisfaction) (to b and c’). All demographic and socioeconomic variables were treated as control variables. If path c from the independent variables and the listed dependent variables was no longer significant after the mediating variable was placed in the model, it indicated a full mediation effect. Otherwise, a partial mediation effect was indicated. Finally, to test the reliability of the mediating effect, the bootstrapping method was further applied to test the significance of direct and indirect effects in the mediating model, with 5,000 random sampling calculations used to obtain the 95% confidence interval (CI) of the estimated value. If the 95% CI of the indirect effect did not contain 0, a mediating effect is considered to exist. STATA/SE 16 (Stata Corp. 2019. Stata Statistical Software: Release 16. College Station, TX) was used to conduct statistical analysis. The significance level of statistical analysis was set at *p* < 0.05 for all analyses. In our study, most variables except gender and residence had more than 0.50% missing data, for instance, “don’t know” and “missing” answers. Specifically, questions about psychological resilience relate to how older adults feel about themselves and may not be judged accurately by others. Due to poor cognitive capacities, approximately 16% of participants above the age of 65 were unable to respond to the questions. Deleting these cases with missing values might bias the results. Based on previous research, multiple imputations (MI) account for the uncertainty of missing data values [69, 70], so MI was conducted using R software version 4.1.2 with the mice package. All missing variables were included in the imputation model, and the number of imputations was 20. The variable distribution of the data after multiple imputation was basically consistent with the data before imputation.

## Results

The distribution features of the research variables are displayed in Table 2. The number of older participants aged 85 years and above reached 52.09%. More than 40% of respondents were male and female, with 12.88% more women than men. Nearly 60% of respondents said they were widowed, never married, or divorced. There was a slightly lower proportion of respondents who received education compared with respondents who had no education (49.55% vs. 50.45%). In terms of current residence, 55.38% of the respondents lived in urban areas. The majority were living with family members (80.39%), whereas 15.93% of respondents lived alone, and only a small proportion were in institutions (3.68%). In addition, most of the participants stated that they perceived their financial support as “enough” (85.60%), while 14.40% considered their financial support to be “not enough”. The proportions of respondents with and without chronic diseases were 69.81% and 30.19%, respectively. The mean scores on low-level involvement activities and high-level involvement activities were 1.796 (SD = 1.324) and 1.329 (SD = 1.219), respectively. The average score of life satisfaction was 3.883 (SD = 0.797), and the mean value of psychological resilience was 19.160 (SD = 3.100).

Table 3 shows the comparison of psychological resilience among older adults with different characteristics. The original scores of psychological resilience variables ranged from 6 to 25. On the basis of a previous similar study [67], the participants were divided into two groups based on the mean psychological resilience scores, namely, those with lower resilience with a score of 19 or less and those with higher resilience with a score of 19 or more. As shown in Table 3, the average level of psychological resilience of rural female participants was lower than that of rural male participants. The average level of psychological resilience of older adults aged ≥ 85 was much lower than that of people aged 65–74 and 75–84. Compared to unmarried and illiterate people, married and literate older adults had a higher level of psychological resilience. Compared to those living alone or in institutions, the average level of psychological resilience was slightly higher for older adults who lived with family. Older adults with enough financial support had a much higher level of psychological resilience.

**Table 3:** Comparison of psychological resilience among older adults with different characteristics

**Characteristics**		Frequency	low-level		high-level		*χ2*	*P value*
			Frequency	*%*	Frequency	*%*		
**Gender**	Male	6873	3107	45.2	3766	54.8	249.014	< 0.001
	Female	8906	5153	57.9	3753	42.1		
**Age**	65–74	3282	1309	39.9	1973	60.1	402.102	< 0.001
	75–84	4277	2061	48.2	2216	51.8		
	≥ 85	8220	4890	59.5	3330	40.5		
**Marital status**	Married	6450	2714	42.1	3736	57.9	461.327	< 0.001
	Windowed, never married and divorced	9329	5546	59.4	3783	40.6		
**Education**	Illiterate	7961	4901	61.6	3060	38.4	546.915	< 0.001
	Literate	7818	3359	43.0	4459	57.0		
**Current residence**	Urban	8738	4419	50.6	4319	49.4	24.756	< 0.001
	Rural	7041	3841	54.6	3200	45.4		
**Living status**	With family	12,684	6496	51.2	6188	48.8	34.334	< 0.001
	Alone	2514	1422	56.6	1092	43.4		
	Institution	581	342	58.9	239	41.1		
**Financial support**	Yes (enough)	13,507	6673	49.4	6834	50.6	325.940	< 0.001
	No (not enough)	2272	1587	69.9	685	30.1		
**Chronic disease**	Yes	11,016	5756	52.3	5260	47.7	0.137	0.711
	No	4763	2504	52.6	2259	47.4		

Notes: *, **, *** Represents a significance level of *p* < 0.05; *p* < 0.01; *p* < 0.001, respectively

### The correlation between social participation, psychological resilience and life satisfaction

Table 4 divided social participation into two levels, low-level involvement activities and high-level involvement activities, to examine correlations among the variables for magnitude and plausibility with regard to our hypothesis and to discuss the impact of each level on life satisfaction when the mediator was psychological resilience. In a comparison of correlations of low-level involvement activities to psychological resilience (*r* = 0.246***, *p* < 0.001), the correlation of low-level involvement activities to life satisfaction was much lower (*r* = 0.082***, *p* < 0.001). High-level involvement activities (*r* = 0.253***, *p* < 0.001; *r* = 0.118***, *p* < 0.001) were associated with psychological resilience and life satisfaction more than low-level involvement activities. There was also a significant relationship between psychological resilience and life satisfaction (*r* = 0.362***, *p* < 0.001). Significant correlations were found between the two levels of social participation and psychological resilience and life satisfaction.

**Table 4:** Correlations(*r*) between social participation, psychological resilience and life satisfaction (*N* = 15,779)

**Variables**	Low-level involvement activities	High-level involvement activities	Psychological resilience	Life satisfaction
Low-level involvement activities	1			
High-level involvement activities	0.557***	1		
Psychological resilience	0.246***	0.253***	1	
Life satisfaction	0.082***	0.118***	0.362***	1

Notes: *, **, *** Represents a significance level of *p* < 0.05; *p* < 0.01; *p* < 0.001, respectively

### The mediating role of psychological resilience between social participation and life satisfaction among older adults in China

With other covariates and the mediating effects of psychological resilience, Table 5 shows the regression model results in the individual analysis of two levels of social participation on life satisfaction. Models 1, 3, 4 and 6 in Table 5 show the dependent variable as life satisfaction, and the dependent variable in Models 2 and 5 is psychological resilience. Model 1 shows a connection between participants’ low-level involvement activities and life satisfaction (*β* = 0.051, *p* < 0.001). Model 2 shows that individuals’ psychological resilience is significantly impacted by their low-level involvement activities (*β* = 0.366, *p* < 0.001). Models 4 and 5 in Table 5 indicate that high-level involvement activities are prominently and actively related to participants’ life satisfaction and psychological resilience (*β* = 0.070, *p* < 0.001; *β* = 0.423, *p* < 0.001; respectively). The results show that high-level involvement activities have a greater effect on the life satisfaction of older adults. Models 3 and 6 show that psychological resilience has a strong positive association with respondents’ life satisfaction.

**Table 5:** The relationship of social participation and life satisfaction and the mediation effects of psychological resilience (*N* = 15,779)

	Low-level involvement activities						High-level involvement activities					
	Model 1: Life satisfaction		Model 2: Psychological resilience		Model 3: Life satisfaction		Model 4: Life satisfaction		Model 5: Psychological resilience		Model 6: Life satisfaction	
	β	S.E	β	S.E	β	S.E	β	S.E	β	S.E	β	S.E
Low-level involvement activities	0.051***	0.006	0.366***	0.022	0.020***	0.005						
High-level involvement activities							0.070***	0.006	0.423***	0.022	0.034***	0.005
Psychological resilience					0.086***	0.002					0.085***	0.002
Sex (ref: Male)	-0.021	0.014	0.345***	0.053	-0.050***	0.013	-0.026	0.014	0.315***	0.053	-0.053***	0.013
Age (ref: 65–74)												
75–84	0.062**	0.018	-0.081	0.070	0.069***	0.017	0.064**	0.018	-0.086	0.070	0.071***	0.017
≥ 85	0.100***	0.020	-0.203**	0.077	0.117***	0.019	0.106***	0.020	-0.229**	0.075	0.125***	0.019
Education (ref: Illiterate)	0.071***	0.015	0.503***	0.056	0.027	0.014	0.069***	0.015	0.521***	0.055	0.024	0.014
Marital status (ref: Married)	0.027	0.016	-0.440***	0.063	0.065***	0.016	0.018	0.016	-0.522***	0.062	0.062***	0.015
Current residence (ref: Rural	0.057***	0.013	0.084	0.048	0.050***	0.012	0.047***	0.013	0.028	0.048	0.044***	0.012
Living status (ref: With family)												
Alone	-0.204***	0.018	-0.117	0.068	-0.194***	0.017	-0.201***	0.018	-0.077	0.068	-0.194***	0.017
Institution	-0.136***	0.033	-0.121	0.127	-0.125***	0.031	-0.152***	0.033	-0.249*	0.126	-0.131***	0.031
Financial support (ref: No)	0.509***	0.018	1.552***	0.067	0.375***	0.017	0.497***	0.018	1.489***	0.067	0.370***	0.017
Chronic disease (ref: No)	-0.014	0.013	-0.266***	0.051	0.009	0.013	-0.016	0.013	-0.274***	0.051	0.007	0.013
N	15,779		15,779		15,779		15,779		15,779		15,779	
R-squared	0.074		0.117		0.173		0.078		0.122		0.174	
Adj. R-squared	0.073		0.116		0.172		0.077		0.121		0.174	

Notes: *, **, *** Represents a significance level of *p* < 0.05; *p* < 0.01; *p* < 0.001, respectively

**Fig. 2:**
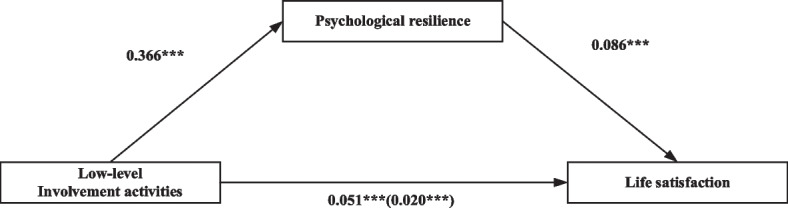
Coefficients estimating Low-level involvement activities → Psychological resilience → Life satisfaction; * = *p* < 0.05, ** = *p* < .01, *** = *p* < .001

**Fig. 3:**
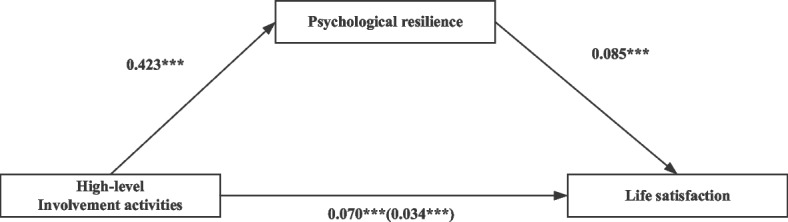
Coefficients estimating High-level involvement activities → Psychological resilience → Life satisfaction; * = *p* < 0.05, ** = *p* < .01, *** = *p* < .001

The mediating role of psychological resilience was tested following Baron et al.’s method [68]. The mediating effects of psychological resilience on the relationship between the two levels of social participation and life satisfaction are depicted in Figs. 2 and 3. Separate mediation models were applied for low-level involvement activities, high-level involvement activities and life satisfaction. As shown in Fig. 2, psychological resilience served as a partial mediator in the link between low-level involvement activities and life satisfaction as the coefficient of social activities decreased from 0.051 to 0.020 after controlling for psychological resilience, and the partial mediating effect size was 0.617. In addition, psychological resilience played a mediating role in the relationship between high-level involvement activities and life satisfaction as the coefficient of high-level involvement activities decreased from 0.070 to 0.034 after controlling for psychological resilience, and the partial mediating effect size was 0.514.

To test the reliability of the mediating effect, our study additionally employed 5000 bootstrapping samples of the raw data [71]. Table 6 lists the direct and indirect impacts of the two levels of social participation on older adults’ life satisfaction along with their corresponding 95% confidence intervals (CIs). The results are shown in Table 6. All CIs did not contain 0, indicating that all paths in the mediated model were significant. The estimated values of the indirect effect were 0.032 and 0.036, respectively, demonstrating that social participation and life satisfaction were mediated by psychological resilience. Furthermore, demographic and sociological characteristics, including age, gender, marital status, degree of education, current residence, living status, financial support, and chronic disease, were correlated with the dependent variables. Therefore, these factors should be controlled during the process of running mediating analyses. According to the relevant literature [72], multicollinearity cannot appear between explanatory variables. In an examination of multicollinearity across all variables, the variance inflation factor (VIF) values ranged from 1.03 to 2.77, which indicated no concerns about multicollinearity.

**Table 6:** Mediation effects of psychological resilience on relationship between social participation and life satisfaction (*N*=15,779)

	Coef	SE	Z	Bias Corrected 95% CI		Percentile 95% CI	
				Lower	Upper	Lower	Upper
**Indirect effects**							
LIA → PR → LS	0.032***	0.002	16.38	0.028	0.035	0.028	0.035
HIA → PR → LS	0.036***	0.002	17.71	0.032	0.040	0.032	0.040
**Direct effects**							
LIA → PR → LS	0.020***	0.005	3.58	0.009	0.030	0.009	0.030
HIA → PR → LS	0.034***	0.006	6.01	0.022	0.044	0.023	0.045

Abbreviations: *LIA* low-level involvement activities, *HIA* high-level involvement activities, *PR* psychological resilience, *LS* life satisfaction

SE Standard Error, CI Confidence Interval, *** *p* < 0.001, ** *p* < 0.01, * *p* < 0.05

## Discussion

This study is novel in its application of a mediation model to measure the mediating role of psychological resilience between social participation and life satisfaction among older adults in China based on activity theory and an active ageing framework. Previous studies have indicated the importance of social participation on older adults’ life satisfaction [73], but research has not confirmed whether psychological resilience can further explain this relationship. This study attempted to identify the relationship between two levels of social participation and the life satisfaction of older adults in China, including low-level involvement activities and high-level involvement activities, with an emphasis on the mediating role of psychological resilience according to the latest wave of the 2018 CLHLS data.

This study found that both levels of social participation were positively correlated with the life satisfaction of older adults in China. A similar study in Chile showed that participation in social participation and life satisfaction were positively correlated among older persons [14]. A Turkish study indicated that restriction in social participation was related to lower life satisfaction [74]. Chinese researchers found that various kinds of social participation had significant positive impacts on life satisfaction. Li demonstrated that a satisfactory life was achieved for more socially active older adults compared to their less socially active counterparts [56]. Social participation involves a certain type of social interaction as well as the realization of self-worth for older adults. As potential ties in real-life social interaction, social participation may help older adults obtain social resources and material goods [75], foster communication and companionship, and improve sociability and social networks in response to the negative effects of physical stressors on people’s well-being [76].

The results in Table 3 demonstrate the comparison of psychological resilience among older adults with different characteristics. There were 52.3% older adults with lower resilience (score ≤ 19), and the average level of psychological resilience of older adults aged ≥ 85 was much lower than those aged 65–74 and 75–84. It is obvious that the psychological resilience of older adults is generally low, and psychological resilience decreases with increasing age. In the process of ageing, older people can face both lifelong adversity and age-specific adversity. Old age is a stage of increased psychosocial stressors, such as death and physical health deterioration [77]. Old age presents more challenges than other life stages; therefore, theories of successful ageing explore positive development in old age by considering these increasing challenges. According to the Tutorial for the Outline of the Healthy China 2030 Plan, strengthening older adults’ health management and promoting the development of mental health for older adults are essential and necessary [78]. A previous study showed that psychological resilience differs with different stages of ageing, different levels of physical health and/or different sexes [63]. Similarly, Neugarten proposed the “standard schedule” of stress events in the life cycle and showed that compared with younger individuals, older individuals aged 80 and above have lower psychological resilience and even more dangerous health risks [79]. The results demonstrated that people who were married, literate, had sufficient financial support and lived with family had higher psychological resilience. The above outcomes suggest that the life satisfaction of older adults living alone is relatively low, which may be due to the desire to maintain interpersonal communication. However, their children are not around them, and they lack sufficient social support; that is, they lack necessary daily life care, spiritual comfort and social participation, leading to loneliness, depression and lower psychological resilience among older adults [80]. Previous research has found that older adults with higher family income and better health status and those who do not live alone have higher psychological resilience [81].

In this study, social participation was classified into two levels, namely, low-level involvement activities and high-level involvement activities. The results of this study showed that each level of social participation was positively correlated with life satisfaction. Specifically, high-level involvement activities were more strongly associated with life satisfaction than low-level involvement activities. The results indicated that older adults may compensate for the lack of personal internal resources to gain life satisfaction through social participation. The ageing of the population inevitably leads to social problems such as a structural shortage of the labour force and an increase in the social dependency ratio and social service, and the rigid retirement system causes a great waste of human resources for older adults. Therefore, the social participation of retired groups is a way to not only develop older adults’ human resources but also to alleviate the shortage of a human resource structure and reduce the social burden with the development of the social economy [82, 83]. From this perspective, society needs to create and reserve a development space for older adults, who should also improve their awareness of social participation.

Our findings identified a partial mediating role of psychological resilience between the two levels of social participation and life satisfaction of older adults in China; that is, social participation indirectly improves the life satisfaction of older adults by influencing their psychological resilience, which also supports the view of successful ageing as the model of selective optimization with compensation [46]. This innovative finding addresses gaps in the research by explaining the relationships among these variables. Resilience has become a major factor in research and mental health theory over the past decades [84]. A previous study reported that insufficient social participation decreases older adults’ life satisfaction, and psychological resilience caused by social isolation deteriorates life satisfaction and increases depressive symptoms [85]. In addition, a previous study regarded resilience as a stable personality trait [86], while recent research has considered it a dynamic process that can change throughout one’s lifetime [87]. The partial mediating role of psychological resilience reveals that other variables may mediate the association between social participation and life satisfaction, such as social support [56].

The results of this study demonstrated that garden work, raising domestic animals, watching TV or listening to the radio, housework and reading newspapers/books are considered low-level involvement activities, while outdoor activities, exercising, travel, playing cards and/or mahjong and organizing social activities are considered high-level involvement activities. High-level involvement activities can help older adults make more friends, improve their interaction abilities, prevent a sense of loss and enhance their self-confidence in integrating into society, thereby helping them to obtain higher psychological resilience and life satisfaction. Outdoor activities, travel, exercising, playing cards and/or mahjong and organizing social activities are the major social communication methods of older adults in both urban and rural areas of China. Additionally, some studies have indicated that engaging in the above activities not only provides opportunities for sufficient physical exercise but also helps develop social networks at the late stage of life for companionship, which is beneficial for emotional and physical health as well as life satisfaction [88]. Health is the cornerstone of life satisfaction, and mental illness and lower psychological resilience are important and are easily neglected. Older adults with mental illness such as anxiety and depression have lower psychological resilience, which may reduce their life satisfaction. However, with the accelerated development of modern society, older adults cannot keep up with the pace of social development and often have a sense of inferiority or loss, which further reduces their psychological resilience. In addition, active theory highlights the partial mediating effects of psychological resilience between social activities and the life satisfaction of older adults [89]. Previous research has indicated that the social participation of older adults exists in the process of social interaction, which is a way of establishing self-esteem, enhancing psychological resilience and achieving resource sharing at the societal level [90]. On the other hand, compared with other Asian areas, older adults in Korea have a gradual decline in their physical and cognitive function as they age, and they often experience reduced engagement in social activities. Therefore, an increasing number of studies have demonstrated the essential role of social participation [91]. For instance, Rochelle used a longitudinal design to demonstrate the importance of social participation to the life satisfaction of older adults in Hong Kong [92]. Other academics have focused on the role of personality in the social activities of older adults and suggested that their personality is greatly affected by their lifestyle in middle age [93]. Murayama et al. believe that, from the perspective of social support theory, access to social resources is related to social status, so older adults with unequal status have inequality in social resources [94]. Therefore, high-level involvement activities can strengthen older adults’ self-esteem and psychological resilience and improve their value capital to maintain their dominant status in social resource exchange and even improve their life satisfaction [95].

Based on the classification of the study, gardening, raising domestic animals, watching TV or listening to the radio, housework and reading newspapers/books were considered low-level involvement activities, which positively influenced older adults’ psychological resilience and life satisfaction. First, compared to high-level involvement activities, activities such as gardening, raising domestic animals, watching TV or listening to the radio, housework and reading newspapers/books entail lower involvement with other individuals. Older adults can also enhance their psychological resilience and life satisfaction by participating in these low-level involvement activities. For instance, they can obtain useful information from their families and friends by doing gardening and housework. Older adults should be encouraged to do housework, such as grocery shopping, cooking and babysitting grandchildren, which are useful types of social participation. Moreover, in the process of housework activities, older adults can achieve personal value and social value in old age. To some extent, the housework activities of older adults can also be a way to enhance psychological resilience and life satisfaction. In addition, older adults can acquire various kinds of knowledge and information and understand the changes and development of society and the community by reading to enrich their awareness, increase their psychological resilience and improve their life satisfaction. Fundamentally speaking, the social participation of older adults is a cultural behaviour, and culture and psychological resilience are the core components that affect older adults’ life satisfaction [96]. In China, older adults’ willingness to learn is not strong enough. Some serious problems exist, such as low education level, poor health and weak participation, with the exception of male, younger and well-educated older adults who have a stronger willingness to participate in learning activities [97]. Younger and literate older adults are more willing to participate in learning activities, which can also increase their psychological resilience. The results showed that half of the older adults were illiterate, which demonstrates the necessity of strengthening older adults’ learning abilities to meet the requirements of social development. Moreover, the learning activities of older adults, such as reading books and newspapers, reflect not only the concept of lifelong learning but also the requirements of active ageing. Currently, senior colleges for older adults are mainly established in urban areas and are less present in rural areas of China. Older adults can acquire skills through senior colleges in addition to common sense and nonskilled knowledge. Reading newspapers and books or studying at senior colleges can greatly strengthen their psychological resilience and enhance their happiness and satisfaction in life [98].

In summary, this study has the following implications. First, both levels of social participation positively impact older adults’ psychological resilience and life satisfaction, although the impact of the two levels of activities is different. The two levels of social participation develop personal value and social value for older persons by influencing older adults’ psychological resilience and life satisfaction. Promoting older adults’ willingness towards social participation may increase life satisfaction directly and indirectly through psychological resilience. In addition, community and social organizations can be more active in establishing older adults’ community life. Communities can develop learning entertainment facilities and space to allow older adults to participate in reading, and social organizations can develop recreational groups that older people can join to have more interactions with others. Second, our study explored the mediating role of psychological resilience as a new underlying mechanism between two levels of social participation and older adults’ life satisfaction in China. This may have practical and psychoeducational implications as it enlarges the scope of intervention in improving life satisfaction. A recent literature review showed that psychological resilience training programmes seem to improve individuals’ well-being across various groups [99]. Thus, developing psychological resilience could also be effective in improving older adults’ life satisfaction. The education system should be more active in fostering resilience to promote life satisfaction. Community and social organizations can play a critical role in establishing mental health services for older persons accessing formal mental health support [100], which may stimulate older adults’ psychological resilience. The support of mental health professionals is likely to reverse negative beliefs about adversity to help older adults be more positive. Moreover, social participation interventions provide a practical means to improve psychological resilience and personality characteristics such as optimism, patience and zest, with an improvement in psychological resilience that contributes to a higher level of life satisfaction. Further study is necessary to deepen our knowledge of the effectiveness of interventions.

In terms of the current study’s limitations, although we used the 2018 wave of cross-sectional data from the CLHLS, we cannot determine a causal inference due to data limitations. Future research should explore the impacts of psychological resilience across subsequent periods of time. Furthermore, we could only use a five-dimensional measure of psychological resilience based on secondary data. However, the five single items used to measure psychological resilience in older adults have been applied elsewhere [63]. Finally, we only discussed the suspected mediation of psychological resilience in social participation and life satisfaction, but the possibility of moderation by psychological resilience also exists. This problem invites further investigation. Despite the limitations mentioned above, our study may contribute to gaining a better understanding of the function of psychological resilience in the lives of older individuals.

## Conclusion

Research has demonstrated that older adults participate in two levels of social activities, and each has a substantial impact on psychological resilience and life satisfaction. In addition, psychological resilience acts as a mediating factor between social participation and life satisfaction among older adults. Psychological resilience reflects the ability of individuals to effectively cope with adversity and loss. In the future, more opportunities are necessary for older adults to increase their social participation. For instance, the community can organize older adults to participate in entertainment activities to enrich their life, entertain their body and mind, increase their knowledge, strengthen their social adaptability, alleviate their psychological helplessness and loneliness, reduce their social isolation, and enhance their psychological resilience and life satisfaction in their later years. This study clarifies the impact of social participation on older adults’ life satisfaction and may also provide evidence for policy-makers and interventionists in formulating policies and creating intervention programmes for older adults, such as eliminating barriers to social participation, establishing a combination of medicine and nursing, providing financial support for older adults’ social organizations and investing more resources in this specific group, to accumulate and maintain psychological resilience.

## Data Availability

This study used the data from the 2018 wave of the Chinese Longitudinal Healthy Longevity Survey (CLHLS), which was investigated by the Peking University. Questionnaires and the datasets can free to download and obtain after users were approved by the agreement of the CLHLS team. https://opendata.pku.edu.cn/dataset.xhtml?persistentId=doi:10.18170/DVN/WBO7LK. If someone wants to request the data from this study, please contact First author (zhiliu0603@163.com) or Corresponding author (hezhifei@swupl.edu.cn).

## Abbreviations

CLHLS: Chinese longitudinal Healthy Longevity Survey
CI: Confidence interval
MI: Multiple imputation
SOC: Selection, optimization, and compensation
